# Enhancing Curcumin’s therapeutic potential in cancer treatment through ultrasound mediated liposomal delivery

**DOI:** 10.1038/s41598-024-61278-x

**Published:** 2024-05-07

**Authors:** Remya Radha, Vinod Paul, Shabana Anjum, Ayache Bouakaz, William G. Pitt, Ghaleb A. Husseini

**Affiliations:** 1 Department of Chemical and Biological Engineering, American University of Sharjah, Sharjah, UAE; 2 Material Science and Engineering PhD Program, College of Arts and Sciences, American University of Sharjah, Sharjah, UAE; 3 UMR 1253, iBrain, Université de Tours, Inserm, Tours, France; 4 Department of Chemical Engineering, Brigham Young University, Provo, UT 84604 USA

**Keywords:** Curcumin liposome, Cytotoxicity, Drug targeting, Microbubbles, Ultrasound triggered release, Fluorescence, Apoptosis, Cancer, Drug discovery

## Abstract

Improving the efficacy of chemotherapy remains a key challenge in cancer treatment, considering the low bioavailability, high cytotoxicity, and undesirable side effects of some clinical drugs. Targeted delivery and sustained release of therapeutic drugs to cancer cells can reduce the whole-body cytotoxicity of the agent and deliver a safe localized treatment to the patient. There is growing interest in herbal drugs, such as curcumin, which is highly noted as a promising anti-tumor drug, considering its wide range of bioactivities and therapeutic properties against various tumors. Conversely, the clinical efficacy of curcumin is limited because of poor oral bioavailability, low water solubility, instability in gastrointestinal fluids, and unsuitable pH stability. Drug-delivery colloid vehicles like liposomes and nanoparticles combined with microbubbles and ultrasound-mediated sustained release are currently being explored as effective delivery modes in such cases. This study aimed to synthesize and study the properties of curcumin liposomes (CLs) and optimize the high-frequency ultrasound release and uptake by a human breast cancer cell line (HCC 1954) through in vitro studies of culture viability and cytotoxicity. CLs were effectively prepared with particles sized at 81 ± 2 nm, demonstrating stability and controlled release of curcumin under ultrasound exposure. In vitro studies using HCC1954 cells, the combination of CLs, ultrasound, and Definity microbubbles significantly improved curcumin’s anti-tumor effects, particularly under specific conditions: 15 s of continuous ultrasound at 0.12 W/cm^2^ power density with 0.6 × 10^7^ microbubbles/mL. Furthermore, the study delved into curcumin liposomes’ cytotoxic effects using an Annexin V/PI-based apoptosis assay. The treatment with CLs, particularly in conjunction with ultrasound and microbubbles, amplified cell apoptosis, mainly in the late apoptosis stage, which was attributed to heightened cellular uptake within cancer cells.

## Introduction

While chemotherapy remains a primary treatment option for most cancers, its severe adverse side effects, increased drug resistance, and general cytotoxicity present significant challenges to its efficacy^1–4^. However, novel drug delivery systems (DDS) are proving useful in enhancing the accumulation of therapeutic drugs in cancerous tissues, prolonging circulation time, and improving therapeutic efficiency^5^. Among these DDS, highly-compatible nanoformulations, such as nanoemulsions and micelles that encapsulate specific therapeutic agents, have shown great promise in site-specific tumor therapies^6^. Unlike traditional medication, nanoparticles have fewer side effects and offer potential solutions to the challenge of multidrug resistance in cancer cells. In recent years, the search for even more efficient nano-vesicular systems, such as liposomes, has increased significantly due to their high biocompatibility indices, low immunogenicity, biosafety, stability, and capacity for targeted delivery^7–9^.

Liposomes are versatile nanoscale carriers composed of bilayered phospholipid vesicles with a large surface-to-volume ratio and an aqueous core capable of compartmentalizing hydrophilic and hydrophobic drugs, as well as other molecules like proteins, sterols, and carbohydrates^10–12^. The controlled release of drugs to the target tissue over an extended period is a significant advantage of liposomal encapsulation technology, as liposomes can retain the encapsulated drugs until disrupted^3,13^. Medical experts widely accept liposomal encapsulation technology as a tissue-specific drug delivery method, since it forms a protective barrier around the drug, protecting it from redox or other chemical reactions and physical binding interactions in the human body^14^. Although chemotherapeutic drugs like Docetaxel, Doxorubicin, Daunorubicin, Cytarabine, Vincristine, Topotecan, Irinotecan, 5-Fluorouracil, Teniposide, Cisplatin, Cladribine, Paclitaxel, and Nelaramine have better treatment efficiency, their severe side effects in patients remain a significant concern^15–18^. In an effort to reduce the undesirable side effects of chemotherapeutic drugs, researchers are turning to the investigation of herbal agents.

Curcumin, a natural bright yellow spice derived from the rhizome of *Curcuma longa Linn*, is a commonly studied compound with two phenolic hydroxyl groups and an enol from a β-diketone moiety^19,20^. The highly pleiotropic curcumin molecule is an active modulator of intracellular signaling pathways that control cell growth and inflammation. Its ability to induce apoptosis in cancer cells without cytotoxic effects on healthy cells suggests it has significant anticancer potential^21,22^. Numerous scientific studies have investigated the beneficial pharmacological effects of curcumin, including its antioxidative, anti-microbial, anti-inflammatory, and anticancer activities, leading to its selection as a safe drug for the prevention and treatment of a wide range of diseases^23–26^. Conversely, the clinical effectiveness of curcumin is limited by poor oral bioavailability, low water solubility, rapid degradation in alkaline conditions, and slow degradation in acidic conditions, among other factors^27^. To overcome these challenges, researchers are focusing on the use of drug delivery vehicles such as liposomes to protectively encapsulate curcumin and improve its efficacy in the body^28^.

The underlying principles behind the design of nanoparticles for delivering drugs to treat cancer are rooted in the concept of the enhanced permeability and retention (EPR) effect^29,30^. In the context of cancer, malignant tumors are characterized by their rapid growth and the subsequent disorganized and permeable nature of their newly formed blood vessels, often lacking robust basement membranes^31^. This disarray in the tumor’s vascular structure creates opportunities for nanoparticles to escape capillaries and penetrate the tumor tissue. Furthermore, due to the inadequate lymphatic drainage within tumors, nanoparticles tend to accumulate and remain concentrated at the tumor site^32^. To exert precise control over when and where drugs are released, researchers have explored various intrinsic and extrinsic triggers. One extensively investigated method is ultrasound, which is widely used in the medical field and generally has no side effects^33,34^. When combined with nano-drug delivery systems, ultrasound has been found to have a synergistic therapeutic effect. This occurs because ultrasound pressure waves can induce both thermal and mechanical effects at the targeted location. Specifically, mild acoustic hyperthermia can enhance the EPR effect by improving vascular permeability^35^.

Microbubbles have emerged as efficient aids for the activated delivery of drugs and genes, along with their utility as contrast enhancers in diagnostic scenarios^36^. Recent investigations have unveiled an intriguing synergy between microbubbles and focused ultrasound, which can induce temporary and reversible openings in the blood–brain barrier non-invasively^37^. Microbubbles, originally developed for contrast-enhanced ultrasound imaging, can induce transient cavitation when exposed to high-intensity ultrasound. This can lead to the permeabilization of the vascular endothelium and further enhance vascular permeability^38,39^. The mechanism of ultrasound-induced microbubble cavitation also facilitates the process of sonoporation, which involves the creation of temporary openings, or pores, in the cell membranes or the vessel wall. These acoustically induced pores allow for rapid drug extravasation and uptake by the cancer cells within the targeted tissues^33,40^.

Extensive research has been conducted on the use of ultrasound to enhance the entrapment, recovery, and formulation of curcumin drugs^41–44^. While ultrasound holds promise for improving curcumin’s efficacy, especially in treating cervical carcinoma, it simultaneously poses a challenge in preserving curcumin’s bioavailability^45^. Furthermore, Yan et al.^46^ research suggests that low-intensity ultrasound could play a crucial role in facilitating the delivery of curcumin to the brain, potentially benefiting individuals with Parkinson’s disease. The use of Lecithin nanoemulsions and other nanoparticles as curcumin carriers for tumor treatment has also been explored^47^. Despite these advancements, the primary challenge persists: finding the optimal equilibrium between maximizing therapeutic effectiveness with ultrasound and maintaining the bioavailability of curcumin. Notably, our study presents a novel approach utilizing FDA-approved liposomes for curcumin encapsulation, distinguishing them from alternative methods such as nanoemulsions. We highlight the innovative use of ultrasound and microbubbles for releasing curcumin from liposomes, which significantly enhances the drug release and its cytotoxic effects on breast cancer cell lines. Through our investigation, we synthesized curcumin-loaded liposomes and optimized both ultrasound-mediated drug release and uptake by HCC 1954 breast cancer cells. This research opens new avenues for enhancing the efficacy of curcumin as a potential therapeutic agent for breast cancer treatment.

## Materials and methods

### Chemicals and cell lines

Curcumin was purchased from Sigma Aldrich. The lipids 1,2-dipalmitoyl-sn-glycerol-3-phosphocholine (DPPC), cholesterol, and 1,2-diastearoyl-sn-glycero-3-phosphoethanolamine-N-[methoxy(polyethylene glycol)-2000] (DSPE–PEG (2000)) were purchased from Avanti Polar Lipids, Inc. (Alabaster, AL, USA) and supplied by Labco LLC in Dubai, UAE. Polycarbonate filters with a pore size of 200 nm and filter supports from Nucleopore Track-Etch membrane filtration products, phosphate-buffered saline (PBS) (pH 7.4), MTT (3-(4,5-dimethyl thiazolyl)-2,5-diphenyl-tetrazolium bromide), and DMSO were bought from Sigma Aldrich and supplied by Labco LLC in Dubai, UAE. Definity microbubbles (1.2 × 10^10^ bubbles/mL, 1.1–3.3 µm in diameter) were procured from Lantheus Medical Imaging and supplied by Labco LLC in Dubai, UAE. All other chemicals used were of high analytical grade and were acquired from Sigma-Aldrich/Merck and supplied by Labco LLC in UAE. The breast cancer cell line HCC1954 was purchased from Addexbio (San Diego, CA, USA), and Mylar sheets (0.1905 mm blank clear plastic sheets) were purchased locally through Amazon.

### Instrumentations

The synthesis and characterization of curcumin liposomes involved several steps and equipment. A Stuart rotary evaporator (RE300) was used to initiate the synthesis process. To obtain unilamellar vesicles, the multilamellar liposomes were subjected to sonication using an Elma D-78224 sonication bath (Melrose Park, IL, USA). Afterward, extrusion with an Avanti Polar Lipids extruder (Alabaster, USA), resulted in uniform-sized curcumin liposomes (CL). The curcumin liposomes' size and other structural properties (CL) were analyzed using the dynamic light scattering method with a Dyanopro Nanostar instrument (Wyatt Technology Corp., Santa Barbara, CA, USA). Free curcumin and CL's fluorescence emission and excitation behavior were quantified using an FLSP920 fluorescence spectrometer (Edinburgh Instruments Ltd., Livingston, UK).

Absorbance measurements were made using a UV–visible spectrophotometer (EVOLUTION 60 S, Thermo Scientific, Waltham, Massachusetts, USA). In vitro studies involving 1 MHz ultrasound delivery employed a Precision Acoustics Ultrasonic transducer, continuous mode (Transducer Diameter 2.54 cm, surface area 1.5 cm^2^) (Dorchester, UK). The Accu Reader plate reader was employed to read 96-well microtiter plates (Accu Reader Metertech, Taiwan). Flow cytometry analysis was carried out using a Beckman Coulter FC500 flow cytometer (Indianapolis, United States).

### Preparation of curcumin liposomes

Curcumin nanoliposomes were prepared using a modified version of the conventional film hydration method with the assistance of a rotary evaporator^8,48^. Initially, DPPC, DSPE-PEG (2000)-NH_2_, and cholesterol were combined in a molar ratio of 13:1:6, along with 1 mg of curcumin, and dissolved in 4 mL of chloroform in a round bottom flask to create a uniform solution. Under vacuum conditions, the chloroform was carefully evaporated using a rotary evaporator at a rotation speed of 120 rpm and 50 °C for 15 min. This process resulted in the formation of a lipid curcumin film at the bottom of the flask. Subsequently, the lipid curcumin film was hydrated with 2 mL of PBS (pH 7.4) while being continuously stirred for 50 min at 60 °C with a magnetic stir bar at 90 rpm. The mixture was sonicated for 2 min in a water bath preset at 60 °C to further enhance the liposome dispersion. The large multilamellar liposome vesicles were extruded by 31 passes at 60 °C through a 200-nm polycarbonate membrane to achieve a homogeneous unilamellar liposomal solution. The next step was purification through a column packed with Sephadex G-100 resin (10 mL) pre-equilibrated with PBS (pH 7.4). Next, the liposomal solution underwent centrifugation (Megafuge 8R centrifuge, Thermo Scientific, USA) at 17,500 rpm for 80 min at 4 °C. The resulting curcumin liposome pellet was washed multiple times with PBS to eliminate any free curcumin. Finally, the washed pellet was suspended in PBS and stored at 4 °C for further analysis.

### Characterization of curcumin liposomes

#### Dynamic light scattering

Liposome size distribution was analyzed using a Dyanopro Nanostar dynamic light scattering instrument (Wyatt Technology Corp., Santa Barbara, CA, USA). To perform the analysis, the curcumin liposome (CL) sample was appropriately diluted in PBS with a pH of 7.4, maintaining a ratio of 1:100 for accurate measurements. Regular assessments were carried out every week to verify the size and stability of the curcumin liposomes under the storage condition of 4 °C. This monitoring ensured the quality and consistency of the liposomal formulation over time.

#### UV–Vis spectroscopy and fluorescence spectroscopy

Spectroscopic analysis was employed to quantify the curcumin liposomes (CL) and free curcumin. Due to curcumin's hydrophobic nature and low water solubility in neutral and acidic conditions^49^, a stock solution of 20 mg/mL of free curcumin in DMSO was utilized. To compare absorbance profiles, the stock solutions of free curcumin and CL were appropriately diluted in PBS (pH = 7.4) and transferred to glass cuvettes, maintaining a consistent curcumin concentration. UV–visible absorption spectra were recorded within a wavelength range of 300–700 nm. To determine the encapsulation efficiency of CL, the liposomal solution was centrifuged at 17,500 rpm at 4 °C, following the procedure outlined in section “Preparation of curcumin liposomes”. The resulting washed pellet was dissolved in DMSO, and the absorbance of curcumin at its maximum wavelength (A_max_ = 432 nm) was measured using the UV–visible spectrophotometer. A calibration curve of free curcumin in DMSO was used to quantify the curcumin content.

Both free curcumin and CL were diluted in PBS with a neutral pH to analyze fluorescence emission profiles. Using a fluorescence spectrometer, fluorescence emission spectra were recorded using an excitation wavelength at 420 nm and capturing emission data within the 435–700 nm range. This analysis provided insights into the fluorescence behavior of both free curcumin and curcumin within the liposomal formulation.

### Stability studies on CL

To ensure the practical suitability of liposomes for drug delivery, it is essential to explore their release kinetics and stability under simulated physiological conditions. So before proceeding to the cell culture studies, the stability of curcumin liposomes (CLs) and the in vitro release of curcumin from the liposomes were assessed. Aliquots of CLs were combined with 3 mL of PBS (pH 7.4) in glass cuvettes and then incubated at 37 °C. At specific time intervals, samples were centrifuged, and the supernatant fraction (20 µL dissolved in DMSO) was analyzed using UV–Vis spectrophotometry; the absorbance at 432 nm was measured to track the release of curcumin. To ensure accuracy and reliability, the study was replicated using three different batches of liposomes, each tested in triplicate.

The percentage release at each time point was calculated based on the relationship below.

1 $$\% release=\frac{{A}_{(t)}-{A}_{(0)}}{{A}_{(f)}-{A}_{(0)}} \times 100\%$$

where, *A*_(*t*)_ is the absorbance value at any time (*t*), *A*_(*0*)_, is the initial absorbance of CLs before incubation at 37 °C, and *A*_(*f*)_, is the absorbance at complete release.

### Ultrasound-mediated release experiments on CL

To investigate the impact of ultrasound on curcumin release from curcumin liposomes (CLs), an initial kinetic study was performed using a Precision Acoustics Ultrasonic transducer (continuous mode operation) operating at 1 MHz. Two sets of CL solutions, each in 3 mL of PBS, were prepared. One set underwent US sonication at an ultrasonic intensity of 0.12 W/cm^2^ for 15 s. The other set, with an equivalent concentration of CLs, without US treatment, served as the control. The % release of curcumin was noted for both control and US-treated fractions and was calculated in a similar way as described in section “Stability studies on CL”.

### In vitro cell culture studies

#### Cell culture and curcumin treatment

Epithelial breast cancer (HCC1954) cell line was procured from the American Type Culture Collection (ATCC, Manassas, VA, USA). The cells were cultured in Roswell Park Memorial Institute (RPMI-1640) media supplemented with 10% Fetal Bovine Serum (FBS 2442, SIGMA ALDRICH), 2 mM glutamine, and antibiotics (penicillin G, 60 mg/L; streptomycin, 100 mg/L). The cells were maintained under proliferation conditions at 37 °C with 5% CO_2_.

For the experimental setup, the cells were seeded into a 12-well plate and exposed to different concentrations of free curcumin, namely 0, 0.5, 1.0, 2.0, and 10 µg/mL. After a 48-h incubation period, the (2.7.2) MTT assay was conducted to evaluate cell viability and assess the effects of curcumin treatment on the HCC 1954 cells.

#### Cytotoxicity studies (MTT assay)

The MTT assay was employed to assess the cytotoxicity of curcumin after treatment with both free and liposomal formulations. Initially, 1 × 10^5^ cells/well were seeded into a 12-well plate and allowed to incubate for 24 h before initiating the treatment. After this incubation period, the cells were exposed to different concentrations of curcumin in fresh media and further incubated for 48 h. Following the 48-h incubation, the cells underwent careful washing twice with PBS (pH = 7.4). Subsequently, MTT (0.5 mg/mL), dissolved in media, was added to all wells, and the plate was incubated at 37 °C for 4 h. After this incubation, DMSO (1 mL) was added to dissolve the formazan crystals that formed in the wells. The optical density (OD) was then measured at 570 nm using a microplate spectrophotometer. As a reference for comparison, wells without curcumin treatment served as control (untreated) wells. The MTT assay was tested with curcumin liposome (CL) with different treatment concentrations and treatment times (24 and 48 h) to confirm the viability at the longer treatment time.

The MTT assay operates on converting the MTT reagent (diphenyl tetrazolium bromide) to formazan, facilitated by metabolically active cells. The production of formazan is proportional to the number of cells and their metabolic activity, integrated over the 4-h exposure of MTT reagent to dehydrogenases in active mitochondria, defining the cell culture's vitality. To calculate cell culture vitality, the ratio of the mean OD values of the treated cells to the mean OD of the control (untreated cells) was determined and then multiplied by 100%. This provided a quantitative assessment of cell viability and the impact of curcumin treatment on the cells in both free and liposomal forms.

Cell culture vitality was calculated by the following relationship:

2 $$\% Culture\, viability=\frac{mean\, absorbance \,of\, test\, wells}{mean\, absorbance \,of\, control}\times 100\%$$

#### Optimization of ultrasound parameters, microbubbles and other conditions for the controlled release of curcumin from CL

**Ultrasound treatment setup**

All the ultrasound treatments on cells were performed within a custom-built water tank (Figure S1), carefully designed to facilitate the treatment of cells positioned on mylar sheets within culture wells. These mylar sheets are acoustically transparent, enabling ultrasound waves to effortlessly penetrate them and effectively reach the cells that were seeded on their surface^50,51^.

A preliminary MTT assay was carried out on healthy breast cancer cell lines to ensure their viability under different ultrasound intensities, thereby optimizing the ultrasound parameters for drug treatment. Subsequently, the cytotoxic activity of curcumin liposomes (CLs) was investigated using the MTT Assay on the HCC 1954 cell line following liposomal treatment and the application of continuous ultrasound at 1 MHz (continuous mode) and different ultrasound intensities (0, 0.07, 0.12, and 0.16 W/cm^2^) and varying exposure times (15, 20, and 30 s). All conditions were tested in triplicates across 3 batches of experiments. After the ultrasound treatment, the standard MTT assay was conducted by incubating the plate for 48 h to assess cell viability and determine the effects of the liposomal treatment in conjunction with the applied ultrasound parameters.

#### Selection of microbubble density/ultrasound for cell viability

To investigate the effects of different ultrasound intensities (0, 0.07, 0.12, and 0.16 W/cm^2^) in combination with 2 different concentrations of Definity (Lantheus, North Billerica, MA, USA) microbubbles (MBs)− 6 × 10^6^ MBs/mL (cells: MBs − 1:30) and 1.7 × 10^7^ MBs/mL (cells: MBs − 1:85), the MTT assay was repeated. Control wells containing untreated HCC 1954 cells (without ultrasound, curcumin, and MBs) were included to establish a baseline for calculating cell viability.

Based on the results of the curcumin MTT assay (section “Cell culture and curcumin treatment”), a curcumin concentration of 2 µg/mL was chosen for further investigation. This concentration was used in conjunction with 15 s of continuous ultrasound at an intensity of 0.12 W/cm^2^ in the presence of 0.6 × 10^7^ MBs/mL (cells: MBs − 1:30). The percentage viability of the cell lines was then calculated and compared with the control conditions to assess the specific combination's impact on cell viability.

### Curcumin uptake studies

To investigate the impact of ultrasound treatment on the cellular uptake of curcumin liposomes (CL), a total of 2 × 10^5^ cells were initially seeded into a 6-well plate coated with mylar sheets and allowed to incubate for 24 h. Subsequently, these cells were subjected to two different conditions: one group received a higher concentration of curcumin liposomes (6 µg/mL) to ensure observable fluorescence, while the other group was exposed to curcumin liposomes in combination with microbubbles for ultrasound treatment. Appropriate control groups were also established. Following the respective treatments, all cells were maintained under identical incubation conditions in the incubator for 1–2 h. To assess the extent of curcumin uptake, the cells were subsequently harvested, pelleted, and resuspended in a PBS buffer solution. The fluorescence analysis was carried out using a Beckman Coulter FC500 flow cytometer (Indianapolis, United States) with curcumin fluorescence detected at 515 nm, using an excitation wavelength of 488 nm.

### Annexin-V/propidium iodide (PI) apoptosis assay

The apoptosis effect of curcumin liposome on HCC1954 cells was evaluated by Annexin V-FITC/PI staining assay kit. Briefly, cells at a seeding density of 3 × 10^5^ were seeded into each well of a 6-well plate loaded with mylar sheets and incubated in the incubator (BIOBASE CO_2_ Incubator, Jinan, China) with 5% CO_2_ maintained at 37 °C. The next day, HCC1954 cells were treated with a curcumin concentration of 2 μg/mL with or without US/MBs with the same optimized conditions, followed by washing the cells with PBS and changing with fresh media containing 10% FBS and incubating for 24 h. The cells were then harvested and washed two times with cold PBS buffer. The cells were resuspended in a 500 µL-binding buffer and stained with fluorescein isothiocyanate (FITC)-labeled Annexin V and propidium iodide (PI) double staining kit (Becton Dickinson, Franklin Lakes, NJ, USA) for 20 min in the dark following the manufacturer’s instruction. Then, flow cytometry was conducted using 20,000 events for each sample (BD FACS Aria III Becton Dickinson, USA). The viable (Annexin−/PI−) and apoptotic cells (Annexin+/PI−; Annexin+/PI+) were quantified using the FlowJo software 10.0.7 (FlowJo LLC, USA). Early apoptosis was characterized by Annexin V+/PI−, and late apoptosis was characterized by Annexin V+/PI+.

#### Statistical analysis

Statistical analysis was performed on the experimental mean data from at least three independent observations. The analyses of data were performed by one-way ANOVA followed by post- Bonferronis Multiple Comparison analysis at a 5% significance level (*p* < 0.05) using a Graph pad Prism 5.0 Software version (San Diego, CA, United States). The standard deviations are shown as error bars ( ±) in respective figures.

## Results

Following the successful synthesis of curcumin-loaded liposomes (CLs), the subsequent steps involved comprehensive structural characterization and quantification of loading efficacy through analyzing their absorbance and emission profiles, and therapeutic efficiency through cell culture studies.

### Characterization of curcumin liposomes

**Figure 1:**
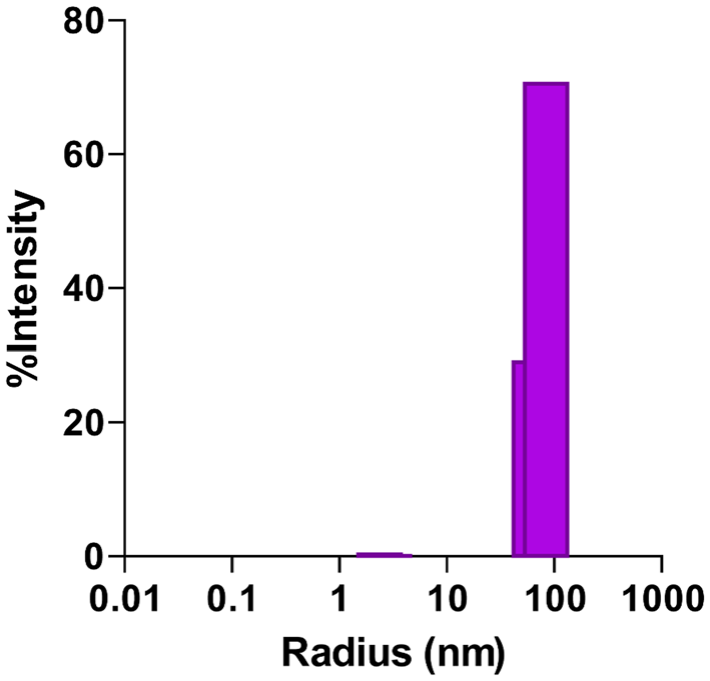
Size distribution of curcumin liposomes by dynamic light scattering.

The nanometer-scale dimensions of the CLs were verified using particle size analysis, as depicted in Fig. 1, revealing a size range of 81.65 ± 2.2 nm. This attribute provides advantageous properties for clinical applications because this size is within the range of the potential enhancements of drug penetration into tissues associated with smaller vesicle particle sizes^52^. The polydispersity index of the vesicles was 0.097 ± 0.0065, underscoring the homogeneity and acceptability of the vesicle population as per the upper limit criterion for % polydispersity of 20% for dynamic light scattering (DLS) measurements. Moreover, these liposomes demonstrated impressive stability, maintaining their attributes over 10 weeks of storage at 4 °C, as substantiated by size and polydispersity measurements (mean radius of 83.5 ± 1.9 nm and polydispersity index of 0.093 ± 0.014).

#### Emission and absorption properties

**Figure 2:**
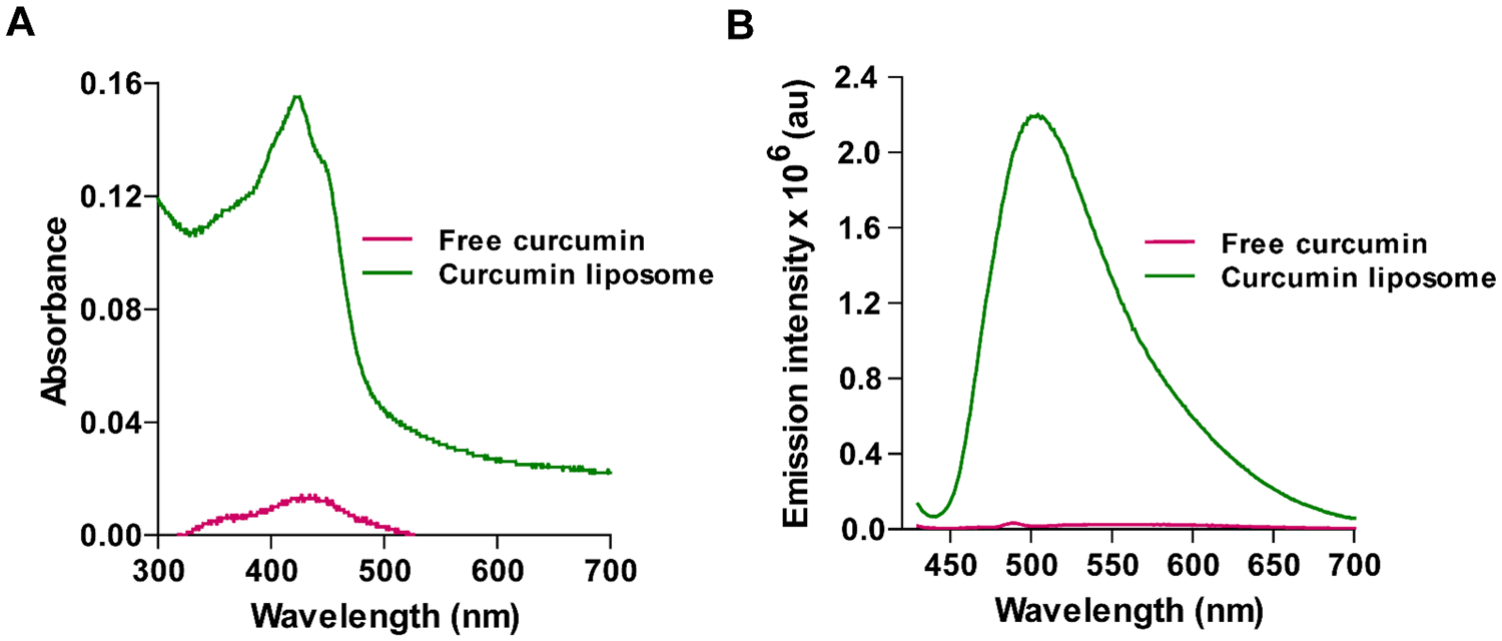
Absorbance and Emission profiles. (**A**) Absorbance spectra of free curcumin (lower pink spectra) and curcumin liposome (upper green plot). (**B**) Emission spectra of free curcumin (lower pink spectra) and curcumin liposome (green plot), Ex-420, Em- 435–700 nm (with 1.5 µg/mL) of curcumin.

To validate curcumin’s attachment to the liposomes, we measured the absorbance and emission profiles of the CLs. UV−vis spectra (Fig. 2A) revealed the curcumin presence in CLs, exhibiting a well-defined peak at 424 nm in PBS (pH 7.4)^53^. Quantitative assessment of curcumin encapsulation efficiency in CLs yielded approximately 33 ± 2%, calculated based on the total curcumin content employed in the liposome synthesis.

The fluorescence emission patterns (Fig. 2B) exhibited a peak emission intensity of 2.2 × 10^6^ arbitrary units (a.u.) at 504 nm.

**Figure 3:**
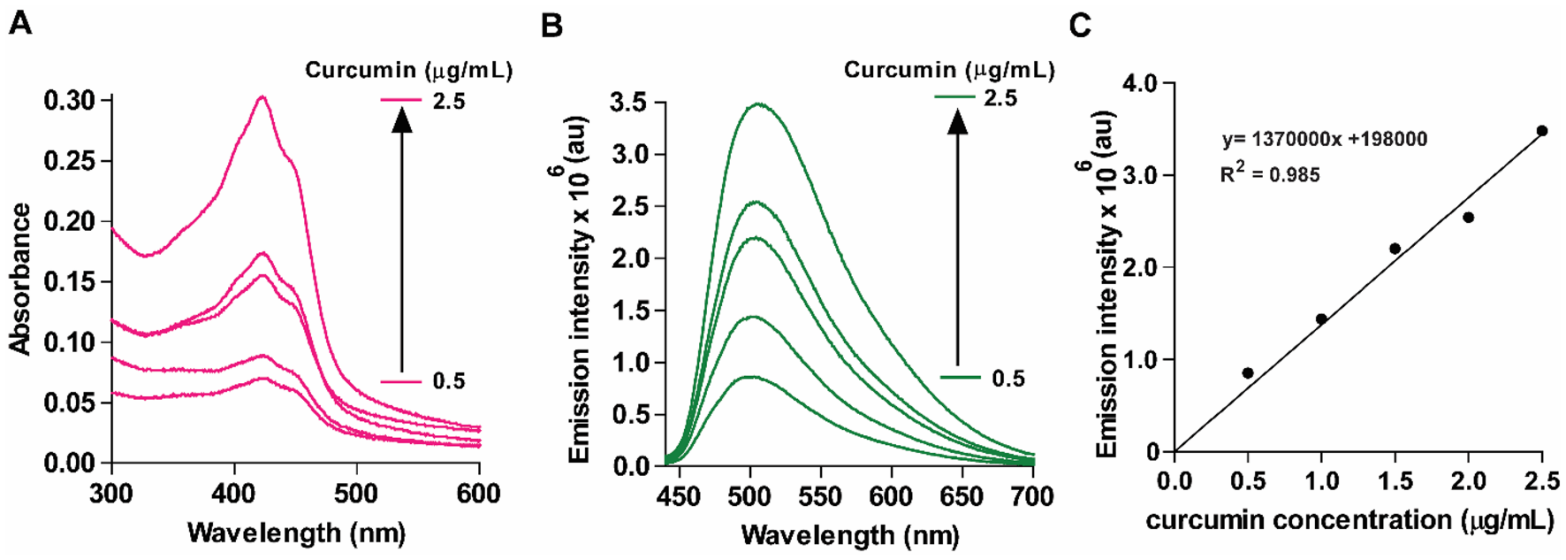
Effect of liposomal curcumin on Absorbance and Emission properties. (**A**) Absorbance spectra for liposomal curcumin for 0.5, 1.0, 1.5, 2.0 and 2.5 µg/mL. The absorption spectra of CL showed an increase in the absorption peaks of curcumin at 424 nm. (**B**) Emission spectra for CL at the wavelength range of 435–700 upon excitation at 420 nm. (**C**) Plot of maximum fluorescence emission intensity (at 504 nm) versus the concentration of curcumin (0.5–2.5 µg/mL).

Strong interaction between curcumin and CLs was reinforced by analyzing UV-absorbance (Fig. 3A) and emission profiles at various CL-curcumin concentrations (ranging from 0.5 to 2.5 µg/mL) (Fig. 3B), revealing linear proportional increases in fluorescence intensity that correspond to liposomal curcumin concentration (Fig. 3C), showing R^2^ value of 0.985. These observations collectively underscore a robust interaction between curcumin and CLs.

### Stability and release studies on curcumin liposomes (CLs)

**Figure 4:**
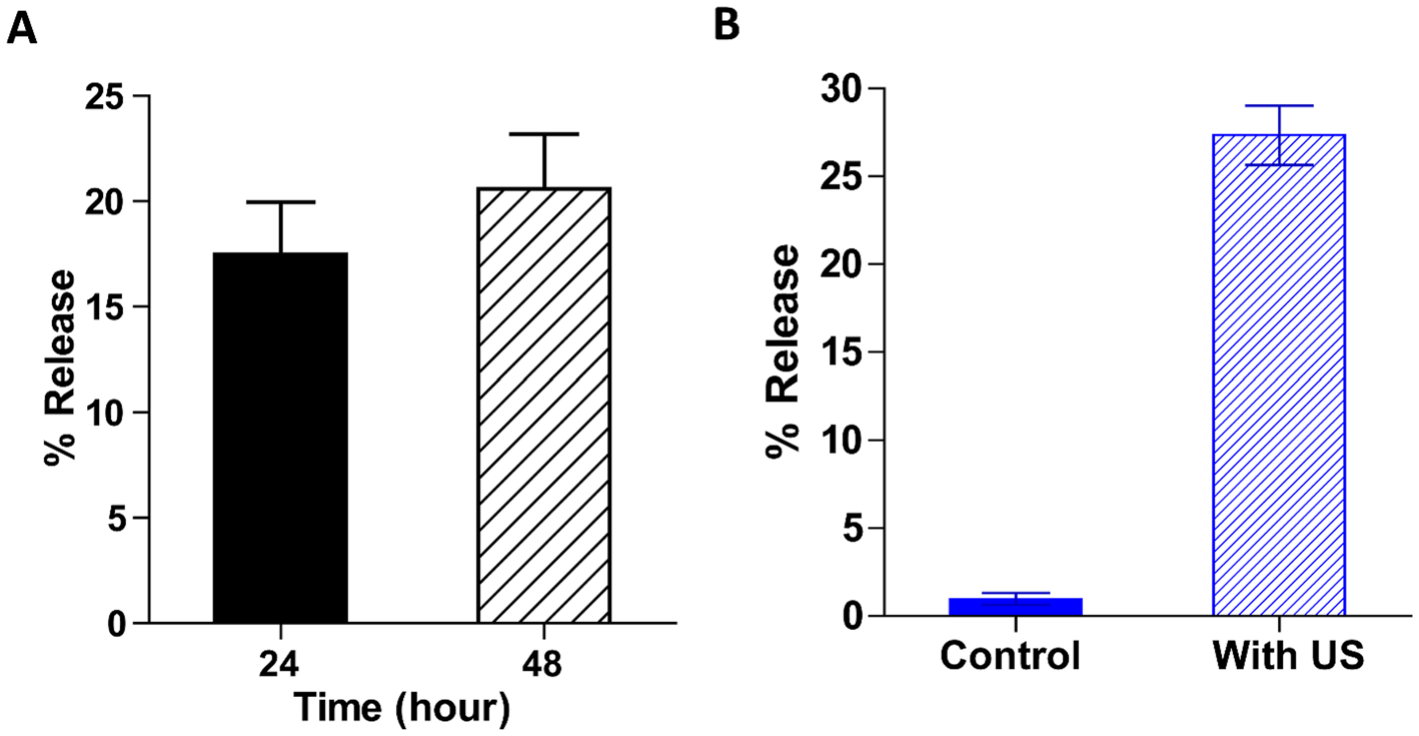
Stability and Ultrasound mediated release studies. (**A**) Stability studies at CLs at 37 °C. The data points represent mean ± standard deviation values from triplicate readings for three different sets of experiments (n = 9). (**B**) In ultrasound-mediated release studies, the percentage of curcumin release from CLs following exposure to the US was at 1 MHz. The bar plots showed the ultrasound-mediated % release for the CLs upon US treatment with MBs with respect to the control fraction. Results were reported as mean ± standard deviation (SD).

We then investigated the stability and release kinetics of CLs under physiological conditions. The percentage of curcumin released from CLs in PBS for 48 h at a temperature of 37 °C is depicted in Fig. 4A. This estimation was carried out using the relationship outlined in section “Stability studies on CL”. Remarkably, even when exposed to a temperature of 37 °C for 2 days, our CLs exhibited excellent stability, with spontaneous curcumin release peaking at 20.5 ± 7% after 48 h. This finding underscores the exceptional stability of CLs under simulated physiological conditions. To ensure the reliability of our findings, we conducted this assessment with three distinct CL batches suspended in PBS, and each trial was executed in triplicate. Furthermore, it is noteworthy that CLs demonstrated a relatively similar release of curcumin into PBS compared to liposomes created through alternative methods for release into the blood plasma, as discussed in references^54,55^.

Additionally, the impact of ultrasound on the release of curcumin from CLs was tested through a release experiment, utilizing Precision Acoustics Ultrasonic transducer, at 1 MHz and a power density of 0.12 W/cm^2^ (15 s). The release was assessed by monitoring alterations in the absorbance value corresponding to curcumin release from the liposomes. In Fig. 4B, we compared the release profile of CLs induced by ultrasound with microbubbles to the control fraction, which was not subjected to ultrasound sonication. There was an immediate steep increase in curcumin release (27 ± 2.5%) upon US treatment compared to the non-sonicated group that showed no immediate release. This unequivocally affirms our study’s validation that ultrasound effectively induces the release of curcumin from liposomes, consistent with analogous observations reported for other liposomes, including Doxorubicin liposomes^56^.

### Ultrasound optimization for cell viability

**Figure 5:**
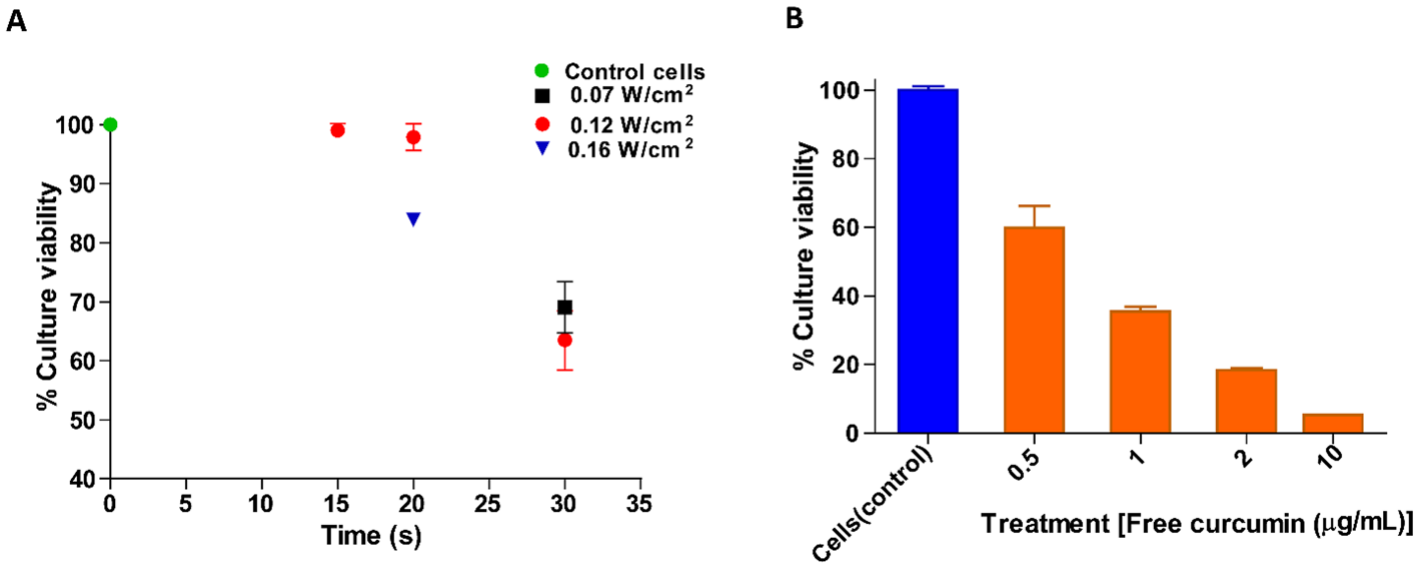
Drug treatment and ultrasound optimization studies. (**A**) Optimization of ultrasound parameters for cell vitality (as per MTT assay). HCC 1954 cells were treated at different power intensities of 0.07, 0.12 and 0.16 W/cm^2^ for varying time at 1 MHz. The results were expressed in % culture vitality for different US treatment conditions. (**B**) The cytotoxic effect of free curcumin on HCC 1954 cells as per MTT assay. The plot indicates the reduction in cell growth upon increasing curcumin concentration. The control has no curcumin. The IC_50_ was approximately 0.68 ± 0.15 µg/mL. The error bars indicate the mean ± standard deviation data of the three different sets of experiments (n = 9).

To ensure the safe application of ultrasound to HCC 1954 cells, we conducted an optimization study to identify the optimum safe acoustic power density for in vitro experiments. This investigation was prompted by concerns related to potential tissue hemorrhage and cell damage when subjecting cultured cells to ultrasound, particularly endothelial cells, as well as potential delicate organs such as the lungs and intestines, to ultrasound exposure^36^. We assessed the viability of HCC cell lines after exposure to different ultrasound intensities (0, 0.07, 0.12, and 0.16 W/cm^2^) at a frequency of 1 MHz, and we varied exposure durations (15, 20, and 30 s) at 37 °C (using the ultrasonication protocol as described in section “Optimization of ultrasound parameters, microbubbles and other conditions for the controlled release of curcumin from CL” and illustrated in supplementary Figure S1). The results of this investigation, as depicted in Fig. 5A, demonstrated a steady decrease in cell viability as the exposure time increased, regardless of the ultrasound intensity. Notably, viability decreased significantly beyond 20 s. These insights culminated in selecting optimal parameters of 0.12 W/cm^2^ intensity and 15 s of exposure for subsequent cytotoxicity and drug uptake analyses.

Figure S2 presents data showing the viability of HCC 1954 cells when exposed to diverse ultrasound intensities (0, 0.07, 0.12, and 0.16 W/cm^2^) alongside varying concentrations of microbubbles. Control groups featuring untreated cells were used to compute cell viability. Our findings reveal that subjecting cells to ultrasound in conjunction with elevated microbubble concentration leads to a notable reduction in cell viability in comparison to the control set. However, cells maintained their viability when exposed to microbubble concentrations lower than 1.5 × 10^7^ microbubbles per milliliter of media. The combination of high-frequency ultrasound and increased microbubble concentrations demonstrated a significant reduction in cell viability.

Studies have reported the advantage of curcumin in preventing carcinogenesis while sparing normal healthy cells^57^. We conducted preliminary MTT tests to investigate the impact of treatment duration on the efficacy of curcumin on HCC 1954 cells. The initial tests were carried out with liposomal curcumin for 24 h and 48 h, but the results indicated that 48 h of treatment (Supplementary Figure S3) were more conclusive than 24 h in showing an effect of concentration. Furthermore, to determine the inhibitory concentration (IC_50_) of free curcumin on these cells, an MTT assay was performed using free curcumin. Figure 5B demonstrates that increased concentrations of free curcumin led to higher toxicity and cell death. The IC_50_ of free curcumin was determined to be ~ 0.68 ± 0.15 µg/mL.

**Figure 6:**
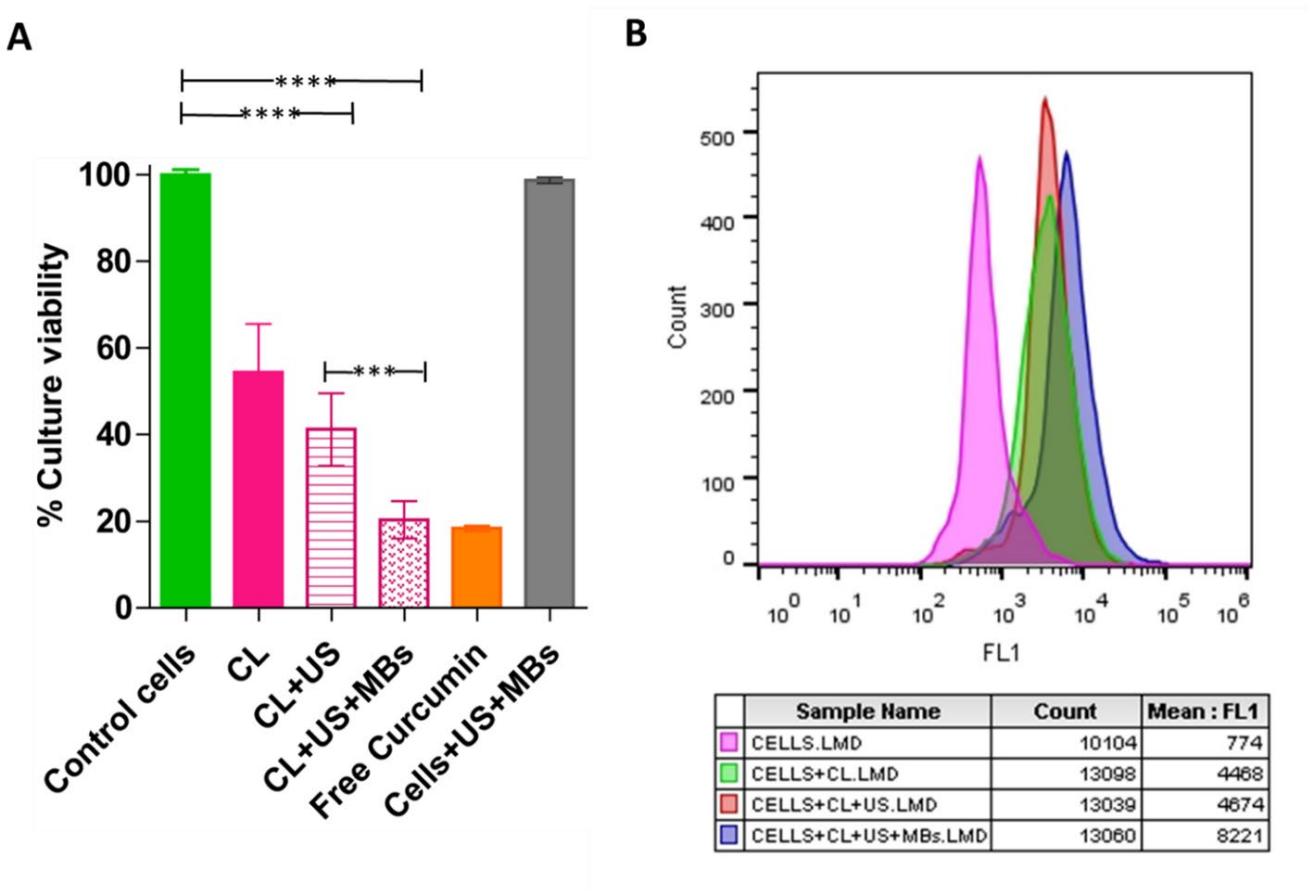
Cytotoxicity and drug uptake analysis. (**A**) MTT assay. Variation in culture viability when cells were treated with CLs for 15 s of continuous ultrasound at a power density of 0.12 W/cm^2^ in the presence of 0.6 × 10^7^ MBs/mL. The bar graph represents different groups: the control cells (representing untreated cells), CL (cells treated with curcumin liposomes), CL + US (cells treated with curcumin liposomes and subjected to ultrasound treatment), CL + US + MBs (cells treated with curcumin liposomes and ultrasound in the presence of microbubbles) and Cells + US + MBs (control cells treated with US in the presence of MBs without curcumin). The height of each bar indicates the percentage of cell viability in the respective treatment groups. The orange bar signifies the percentage of cell viability when the cells were treated with an equivalent quantity of free curcumin, providing a benchmark for comparison with the liposomal treatment groups. The results were statistically significant with ****p < 0.0001 compared to the respective control data set at I = 0 W/cm^2^/without MBs and for the condition Cell + CL + US without the use of MBs estimated using One-way ANOVA. Data in the figure are means ± SD of three independent experiments. (**B**) curcumin uptake analysis by flow cytometry. Cellular uptake of curcumin in HCC 1954 cells post-incubation with curcumin-loaded liposomes (CLs) following ultrasound sonication at 0.12 W/cm^2^ for 15 s. Untreated cells were used as a negative control to establish the baseline. Here, the FL1 corresponds to the fluorescence emission (curcumin) at 515 nm.

Finally, the cytotoxic activity of CLs was investigated using the MTT assay on the same cell lines after exposure to CLs and 15 s of continuous ultrasound (1 MHz) at 0.12 W/cm^2^ in the presence of 0.6 × 10^7^ MBs/mL, as shown in Fig. 6A. At the optimized conditions of ultrasound and microbubbles, the uptake of curcumin by cancer cells was found to increase. With the addition of microbubbles, cell viability further decreased. The treatment with ultrasound and microbubbles was statistically significant with ****p < 0.0001 [compared to the respective control data set without ultrasound treatment/MBs and with cells treated with CLs and ultrasound]. Simultaneously, the utilization of microbubbles demonstrated a significantly higher cytotoxic effect (***p < 0.001) compared to the CL + US group.

The investigation into the uptake of curcumin using curcumin liposomes revealed a noticeable increase in curcumin internalization. The application of ultrasound in combination with microbubbles further amplified this uptake, as depicted in Fig. 6B. The data demonstrate that ultrasound with MBs led to a substantial rise in fluorescent intensity from 4674 to 8221 arbitrary units in FL1, providing compelling evidence that ultrasound with MBs played a crucial role in facilitating the release of curcumin from the liposomes^3^.

**Figure 7:**
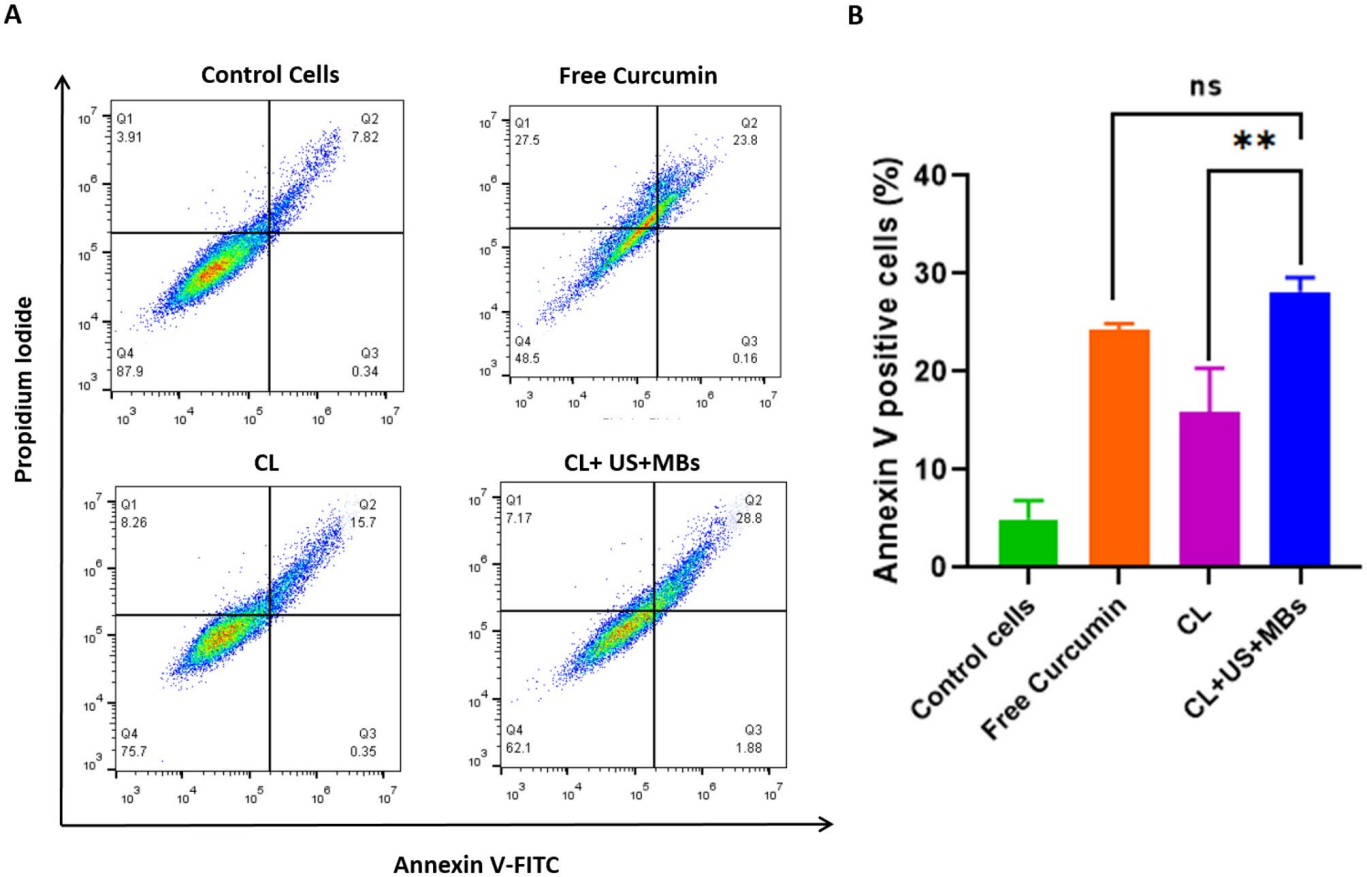
Apoptosis data showing the effect of curcumin liposome on Annexin V and PI staining with or without ultrasound (**A**) Scatter plots representing Annexin V (x-axis) and PI staining (y-axis) in HCC 1954 cancer cell lines with different treatment conditions. (**B**) Bar graph showing the percentage of Annexin V positive cells for different treatments. Here ‘ns’- stands for non-significant.

The efficacy of CLs in exerting an anticancer influence was further verified using an Annexin V/PI-based apoptosis assay. Annexin V is commonly employed as a marker to detect the externalization of phosphatidylserine, a characteristic behavior of cells undergoing apoptosis. Free curcumin displayed the ability to induce either necrotic or apoptotic effects on HCC 1954 cells. Treatment with CLs (2 µg/mL) revealed that a proportion of cells were in the later stages of programmed cell death (Annexin+/PI+) (approximately 16%) compared to untreated cells (shown in Fig. 7). Notably, the administration of CLs in combination with ultrasound and microbubbles led to a substantial increase in cell apoptosis (*p* < 0.001) compared to CL (treatment without US/MBs).

The application of CLs in conjunction with ultrasound significantly resulted in a noteworthy augmentation of cancer cell apoptosis, with a predominant portion of cells occupying the late apoptosis stage. Roughly 30% of cells were observed in this late apoptotic phase, accompanied by a small percentage of necrotic cells (7–10%) (Fig. 7B). This observation underscores the potential cytotoxic impact of our liposomal formulations. The amplified apoptotic effect observed with CLs can be attributed to the heightened cellular uptake within the cancer cells.

## Discussion

The multifaceted properties of curcumin have been the focus of extensive scientific exploration, highlighting its diverse health benefits^24,58^. Nevertheless, the significant challenges of limited bioavailability and its hydrophobic nature present significant barriers to its broader use as a therapeutic or medicinal agent^59^. To fully unlock its potential in addressing various health conditions, particularly in cancer therapy, novel advanced delivery methods are imperative.

Our study introduces an inventive approach to cancer therapy, seamlessly combining nanoliposomes, curcumin, ultrasound, and microbubbles to enhance drug delivery and enhance therapeutic outcomes. The use of liposomes as drug carriers is substantiated by their strong credentials, including high biocompatibility, biodegradability, and amphiphilic nature^7,60^. These attributes make them a compelling choice for advanced drug delivery strategies, and our research effectively underscores the advantages of employing liposomal curcumin.

Due to its remarked hydrophobic nature, curcumin molecules are expected to be enclosed within the nanoliposome’s lipid bilayer region as part of the lipid thin-film formation process, much like other hydrophobic compounds or drugs^61,62^. The strategic integration of curcumin into nanoliposomes is specifically designed to enhance its bioavailability, addressing the challenges posed by its hydrophobic properties^63^.

Our study highlights curcumin-loaded liposomes (CLs) nanoscale dimensions, positioning them as a potentially suitable option for clinical applications. The low polydispersity index and the stability of CLs over time are pivotal factors in ensuring consistent and reliable drug delivery (Fig. 1). This research achieves the successful encapsulation of curcumin within CLs, validated through UV–Vis spectra and fluorescence intensity measurements (Fig. 2). UV−vis spectra revealed a distinct peak at 424 nm in a suspension of CLs in PBS, confirming the presence of curcumin within the liposomes. The quantitative assessment of curcumin encapsulation efficiency, approximately 33 ± 2%, solidifies the successful loading of curcumin in the liposomes. The substantial enhancement in fluorescence intensity, a remarkable 132-fold amplification compared to free curcumin in PBS, indicates that the liposomal formulation significantly improves curcumin's solubility and fluorescence. As reported, curcumin is nearly insoluble in aqueous solution at neutral pH, but the presence of lipids led to a substantial increase in the fluorescence intensity of liposomal curcumin, as clearly illustrated by the stark difference compared to free curcumin. This observation is consistent with previous research findings reported in the literature^64^. The substantial improvement in curcumin's solubility and fluorescence within the liposomal formulation underscores the efficacy of this approach in enhancing curcumin's suitability for therapeutic use.

The controlled release mechanism is crucial in drug delivery systems, ensuring a consistent and predictable release of therapeutic agents^65^. Unlike traditional methods that rely on passive diffusion, ultrasound-mediated drug delivery takes an active approach by targeting tumor cells, enabling precise and controlled drug release while minimizing potential side effects^66^. The introduction of ultrasound and microbubbles for curcumin release constitutes another groundbreaking innovation. The study demonstrates a substantial increase in curcumin release facilitated by ultrasound (Fig. 4b), indicating the exciting possibilities for non-invasive drug delivery^29^.

The research underscores the remarkable cytotoxicity of CLs, especially when combined with ultrasound and microbubbles (Fig. 6). Ultrasound treatment with microbubbles was found to increase the cytotoxic effect of liposomal formulations toward the cell line, possibly due to the enhanced permeability of cellular and liposomal membranes, which can potentially result in high uptake of encapsulated therapeutic agents^35,56^. Comparable investigations have also observed enhanced uptake and release of Doxorubicin and other chemotherapy agents with ultrasound in the presence of microbubbles^67–69^.

The results of uptake and apoptosis studies (Fig. 7) suggest that sonoporation and excitation of MBs create pores in the cancer cells, enhancing the uptake of the drug into the target cells, and pre-clinical studies recommend efficient drug delivery by MBs mediated by liposomes^70,71^. Therefore, microbubble-mediated drug uptake by tumor cells is attributed to direct intratumoral exposure-free drug released upon concurrent exposure of CLs and MBs to high-frequency ultrasound. The increased cellular uptake and the ability to induce apoptosis in cancer cells underscore the promising potential of this formulation as an effective strategy for cancer therapy.

These results represent a substantial leap forward in cancer therapy by harnessing the strengths of nanoliposomes, curcumin, and microbubble-mediated ultrasound-triggered drug release. Notably, both liposomes and microbubbles hold FDA approval as preferred carriers for clinical applications, distinguishing them from alternative nano-carriers such as nanoemulsions^72,73^. In this way, our investigation paves the way for the utilization of liposomal-curcumin formulations to enhance drug delivery efficacy, with ultrasound and microbubbles under optimized conditions. This innovative approach has the potential to transform cancer treatment and stimulate further exploration and development in the field of targeted drug delivery systems.

## Conclusions

In conclusion, this study demonstrates the effective utilization of liposomes as a delivery system for curcumin, thereby offering a promising avenue for targeted breast and other cancer treatments. The liposomal curcumin attachment, ultrasound-mediated controlled release, and improved cellular uptake of curcumin achieved through our curcumin-loaded liposomes (CLs) present exciting prospects for mitigating the side effects of conventional chemotherapy.

Furthermore, our in vitro experiments conducted on HCC1954 cells indicate that the use of CLs, particularly in conjunction with ultrasound and microbubbles, significantly enhances the anti-tumor activity of this herbal therapeutic agent compared to treatments without ultrasound. Ultrasound triggers microbubble oscillation and cavitation, disrupting liposomes and releasing drugs. Due to their small size, microbubbles can efficiently circulate in blood vessels and penetrate tissues under ultrasound treatment. Their oscillation temporarily boosts cell membrane permeability, enhancing drug uptake. This finding highlights the potential of combining liposomal curcumin with ultrasound for enhanced cancer therapy. Moving forward, the next crucial steps involve in vivo applications to assess drug accumulation and measure tumor regression under ultrasound exposure. This critical research phase holds the promise of establishing a more effective platform for delivering herbal drugs in a targeted manner, which could potentially revolutionize cancer treatment strategies. Overall, our study represents a significant step towards developing safer and more efficient approaches for cancer therapy, ultimately benefiting patients in their fight against this devastating disease.

### Supplementary Information

Supplementary Figures.

## Data Availability

The datasets generated and or analyzed during the study are available in Google Drive https://drive.google.com/drive/folders/15Ww7g1XuT4mrTkJqVVOL9_OFatlh-RUZ?usp=drive_link.

## References

[1] Nakatsuji H (2021). Cancer-microenvironment triggered self-assembling therapy with molecular blocks. Mater. Horiz..

[2] Wang X, Zhang H, Chen X (2019). Drug resistance and combating drug resistance in cancer. Cancer Drug Resist..

[3] Elamir A (2021). Ultrasound-triggered herceptin liposomes for breast cancer therapy. Sci. Rep..

[4] Anand U (2022). Cancer chemotherapy and beyond: Current status, drug candidates, associated risks and progress in targeted therapeutics. Genes Dis..

[5] Al Bostami RD, Abuwatfa WH, Husseini GA (2022). Recent advances in nanoparticle-based co-delivery systems for cancer therapy. Nanomaterials.

[6] Sánchez-López E (2019). Current applications of nanoemulsions in cancer therapeutics. Nanomaterials.

[7] Aguilar-Pérez KM, Avilés-Castrillo JI, Medina DI, Parra-Saldivar R, Iqbal HMN (2020). Insight Into nanoliposomes as smart nanocarriers for greening the twenty-first century biomedical settings. Front. Bioeng. Biotechnol..

[8] Radha R, Al-Sayah MH (2021). Development of liposome-based immunoassay for the detection of cardiac troponin I. Molecules.

[9] Radha R, Shahzadi SK, Al-Sayah MH (2021). Fluorescent immunoassays for detection and quantification of cardiac troponin I: A short review. Molecules.

[10] AlSawaftah N, Pitt WG, Husseini GA (2021). Dual-targeting and stimuli-triggered liposomal drug delivery in cancer treatment. ACS Pharmacol. Transl. Sci..

[11] AlSawaftah NM (2022). Ultrasound-sensitive cRGD-modified liposomes as a novel drug delivery system. Artif. Cells Nanomed. Biotechnol..

[12] Tenchov R, Bird R, Curtze AE, Zhou Q (2021). Lipid nanoparticles─from liposomes to mRNA vaccine delivery, a landscape of research diversity and advancement. ACS Nano.

[13] Nsairat H (2022). Liposomes: structure, composition, types, and clinical applications. Heliyon.

[14] Allahou LW, Madani SY, Seifalian A (2021). Investigating the application of liposomes as drug delivery systems for the diagnosis and treatment of cancer. Int. J. Biomater..

[15] Zhao Y (2019). Irinotecan, topotecan, paclitaxel or docetaxel for second-line treatment of small cell lung cancer: a single-center retrospective study of efficiency comparation and prognosis analysis. Transl. Lung Cancer Res..

[16] Goto K (2016). Combined chemotherapy with cisplatin, etoposide, and irinotecan versus topotecan alone as second-line treatment for patients with sensitive relapsed small-cell lung cancer (JCOG0605): A multicentre, open-label, randomised phase 3 trial. Lancet Oncol..

[17] Smorenburg CH, Sparreboom A, Bontenbal M, Verweij J (2001). Combination chemotherapy of the taxanes and antimetabolites. Eur. J. Cancer.

[18] He K, Tang M (2018). Safety of novel liposomal drugs for cancer treatment: Advances and prospects. Chem. Biol. Interact..

[19] Amalraj A, Pius A, Gopi S, Gopi S (2017). Biological activities of curcuminoids, other biomolecules from turmeric and their derivatives–a review. J. Tradit. Complement. Med..

[20] Stanić Z (2017). Curcumin, a compound from natural sources, a true scientific challenge–a review. Plant Foods Hum. Nutr..

[21] Khosropanah MH (2016). Analysis of the antiproliferative effects of curcumin and nanocurcumin in MDA-MB231 as a breast cancer cell line. Iran. J. Pharm. Res. IJPR.

[22] Mukherjee D, Krishnan A (2023). Therapeutic potential of curcumin and its nanoformulations for treating oral cancer. World J. Methodol..

[23] Zhou H, Beevers CS, Huang S (2011). The targets of curcumin. Curr. Drug Targets.

[24] Sharifi-Rad J (2020). Turmeric and its major compound curcumin on health: Bioactive effects and safety profiles for food, pharmaceutical. Biotechnol. Med. Appl. Front. Pharmacol..

[25] Hewlings S, Kalman D (2017). Curcumin: A review of its effects on human health. Foods.

[26] Radha R, Makhlouf Z, Diab R, Al-Sayah MH (2024). Modifying cellulose fibres with carbon dots: A promising approach for the development of antimicrobial fibres. R. Soc. Open Sci..

[27] Dei Cas M, Ghidoni R (2019). Dietary curcumin: Correlation BETWEEN BIOAVAILABILITY AND HEALTH POTENTIal. Nutrients.

[28] Chen Y, Lu Y, Lee RJ, Xiang G (2020). Nano encapsulated curcumin: And its potential for biomedical applications. Int. J. Nanomed..

[29] Mitchell MJ (2021). Engineering precision nanoparticles for drug delivery. Nat. Rev. Drug Discov..

[30] Tian H (2022). Enhancing the therapeutic efficacy of nanoparticles for cancer treatment using versatile targeted strategies. J. Hematol. Oncol..

[31] Liu Z-L, Chen H-H, Zheng L-L, Sun L-P, Shi L (2023). Angiogenic signaling pathways and anti-angiogenic therapy for cancer. Signal Transduct. Target. Ther..

[32] Torchilin V (2011). Tumor delivery of macromolecular drugs based on the EPR effect. Adv. Drug Deliv. Rev..

[33] Pitt WG, Husseini GA, Staples BJ (2004). Ultrasonic drug delivery–a general review. Expert Opin. Drug Deliv..

[34] Awad NS (2021). Ultrasound-responsive nanocarriers in cancer treatment: A review. ACS Pharmacol. Transl. Sci..

[35] Tharkar P, Varanasi R, Wong WSF, Jin CT, Chrzanowski W (2019). Nano-enhanced drug delivery and therapeutic ultrasound for cancer treatment and beyond. Front. Bioeng. Biotechnol..

[36] Tsutsui JM, Xie F, Porter RT (2004). The use of microbubbles to target drug delivery. Cardiovasc. Ultrasound.

[37] Sierra C (2017). Lipid microbubbles as a vehicle for targeted drug delivery using focused ultrasound-induced blood–brain barrier opening. J. Cereb. Blood Flow Metab..

[38] Sun R (2022). The tumor EPR effect for cancer drug delivery: Current status, limitations, and alternatives. Adv. Drug Deliv. Rev..

[39] Duan L (2020). Micro/nano-bubble-assisted ultrasound to enhance the EPR effect and potential theranostic applications. Theranostics.

[40] Man VH (2019). Molecular mechanism of the cell membrane pore formation induced by bubble stable cavitation. J. Phys. Chem. B.

[41] Shirsath SR, Sable SS, Gaikwad SG, Gogate PR (2021). Ultrasound assisted curcumin recovery from Curcuma aromatica: Understanding the effect of different operating parameters. Chem. Eng. Process. Process Intensif..

[42] Selmanovic S (2017). Therapeutic effects of curcumin on ultrasonic morphological characteristics of liver in patients with metabolic syndrome. Acta Inform. Med..

[43] Ahmad N (2019). Preparation of a novel curcumin nanoemulsion by ultrasonication and its comparative effects in wound healing and the treatment of inflammation. RSC Adv..

[44] Wahyudiono (2022). Curcumin-loaded liposome preparation in ultrasound environment under pressurized carbon dioxide. Foods.

[45] Carr KR, Ioffe YJ, Filippova M, Duerksen-Hughes P, Chan PJ (2015). Combined ultrasound-curcumin treatment of human cervical cancer cells. Eur. J. Obstet. Gynecol. Reprod. Biol..

[46] Yan Y (2021). Brain delivery of curcumin through low-intensity ultrasound-induced blood-brain barrier opening via lipid-PLGA nanobubbles. Int. J. Nanomed..

[47] Prasad C, Bhatia E, Banerjee R (2020). Curcumin encapsulated lecithin nanoemulsions: An oral platform for ultrasound mediated spatiotemporal delivery of curcumin to the tumor. Sci. Rep..

[48] AlSawaftah NM (2021). Transferrin-modified liposomes triggered with ultrasound to treat HeLa cells. Sci. Rep..

[49] Zheng B, McClements DJ (2020). Formulation of more efficacious curcumin delivery systems using colloid science: enhanced solubility, stability, and bioavailability. Molecules.

[50] Yoo S, Mittelstein DR, Hurt RC, Lacroix J, Shapiro MG (2022). Focused ultrasound excites cortical neurons via mechanosensitive calcium accumulation and ion channel amplification. Nat. Commun..

[51] Lee K (2023). Ultrasonocoverslip: In-vitro platform for high-throughput assay of cell type-specific neuromodulation with ultra-low-intensity ultrasound stimulation. Brain Stimul..

[52] Barua S, Mitragotri S (2014). Challenges associated with penetration of nanoparticles across cell and tissue barriers: A review of current status and future prospects. Nano Today.

[53] Upadhyay A, Yagnik B, Desai P, Dalvi SV (2018). Microbubble-mediated enhanced delivery of curcumin to cervical cancer cells. ACS Omega.

[54] Mahmud M, Piwoni A, Filiczak N, Janicka M, Gubernator J (2016). Long-circulating curcumin-loaded liposome formulations with high incorporation efficiency, stability and anticancer activity towards pancreatic adenocarcinoma cell lines in Vitro. PLOS ONE.

[55] Saraf S (2020). Advances in liposomal drug delivery to cancer: An overview. J. Drug Deliv. Sci. Technol..

[56] Abuwatfa WH (2022). In vitro evaluation of ultrasound effectiveness in controlling doxorubicin release from albumin-conjugated liposomes. J. Biomed. Nanotechnol..

[57] Liu Z (2018). Preventive effect of curcumin against chemotherapy-induced side-effects. Front. Pharmacol..

[58] Cheng C (2017). Improved bioavailability of curcumin in liposomes prepared using a pH-driven, organic solvent-free, easily scalable process. RSC Adv..

[59] Shen L, Liu C-C, An C-Y, Ji H-F (2016). How does curcumin work with poor bioavailability? Clues from experimental and theoretical studies. Sci. Rep..

[60] Díaz M, Vivas-Mejia P (2013). Nanoparticles as drug delivery systems in cancer medicine: Emphasis on RNAi-containing nanoliposomes. Pharmaceuticals.

[61] De Leo V (2018). Encapsulation of curcumin-loaded liposomes for colonic drug delivery in a pH-responsive polymer cluster using a pH-driven and organic solvent-free process. Molecules.

[62] Joguparthi V, Xiang T, Anderson BD (2008). Liposome transport of hydrophobic drugs: Gel phase lipid bilayer permeability and partitioning of the lactone form of a hydrophobic camptothecin, DB-67. J. Pharm. Sci..

[63] Tabanelli R, Brogi S, Calderone V (2021). Improving curcumin bioavailability: Current strategies and future perspectives. Pharmaceutics.

[64] Moussa Z, Chebl M, Patra D (2017). Fluorescence of tautomeric forms of curcumin in different pH and biosurfactant rhamnolipids systems: Application towards on-off ratiometric fluorescence temperature sensing. J. Photochem. Photobiol. B.

[65] Geraili A, Xing M, Mequanint K (2021). Design and fabrication of drug-delivery systems toward adjustable release profiles for personalized treatment. VIEW.

[66] Adepu S, Ramakrishna S (2021). Controlled drug delivery systems: Current status and future directions. Molecules.

[67] Yu FTH, Chen X, Wang J, Qin B, Villanueva FS (2016). Low intensity ultrasound mediated liposomal doxorubicin delivery using polymer microbubbles. Mol. Pharm..

[68] Wang C (2023). Ultrasound-responsive low-dose doxorubicin liposomes trigger mitochondrial DNA release and activate cGAS-STING-mediated antitumour immunity. Nat. Commun..

[69] Bahutair WN, Abuwatfa WH, Husseini GA (2022). Ultrasound triggering of liposomal nanodrugs for cancer therapy: A review. Nanomaterials.

[70] Ingram N (2020). Ultrasound-triggered therapeutic microbubbles enhance the efficacy of cytotoxic drugs by increasing circulation and tumor drug accumulation and limiting bioavailability and toxicity in normal tissues. Theranostics.

[71] Prabhakar A, Banerjee R (2019). Nanobubble liposome complexes for diagnostic imaging and ultrasound-triggered drug delivery in cancers: A theranostic approach. ACS Omega.

[72] Kenchegowda M (2021). Smart nanocarriers as an emerging platform for cancer therapy: A review. Molecules.

[73] Wu S-K, Tsai C-L, Huang Y, Hynynen K (2020). Focused ultrasound and microbubbles-mediated drug delivery to brain tumor. Pharmaceutics.

