# Supplementary material for: Enhancing Curcumin’s therapeutic potential in cancer treatment through ultrasound mediated liposomal delivery
Source: Sci Rep. 2024 May 7;14:10499. doi: 10.1038/s41598-024-61278-x (PMC11076529; doi:10.1038/s41598-024-61278-x)
Supplement: Supplementary file 1 — Supplementary Figures. [file 41598_2024_61278_MOESM1_ESM.docx]

**Enhancing Curcumin's Therapeutic Potential in Cancer Treatment through Ultrasound-Mediated Liposomal Delivery**

Remya Radha^1^, Vinod Paul^1,2^, Shabana Anjum^1^, Ayache Bouakaz^3^, William G. Pitt^4^, Ghaleb A. Husseini^1,2,*^

^1^Department of Chemical and Biological Engineering, American University of Sharjah, Sharjah, UAE

^2^Material Science and Engineering PhD program, College of Arts and Sciences, American University of Sharjah, Sharjah, UAE

^3^UMR 1253, iBrain, Université de Tours, Inserm, Tours, France

^4^Department of Chemical Engineering, Brigham Young University, Provo, UT, 84604, USA

*corresponding author: [ghusseini@aus.edu](mailto:ghusseini@aus.edu)

**SUPPLEMENTARY DATA**

**
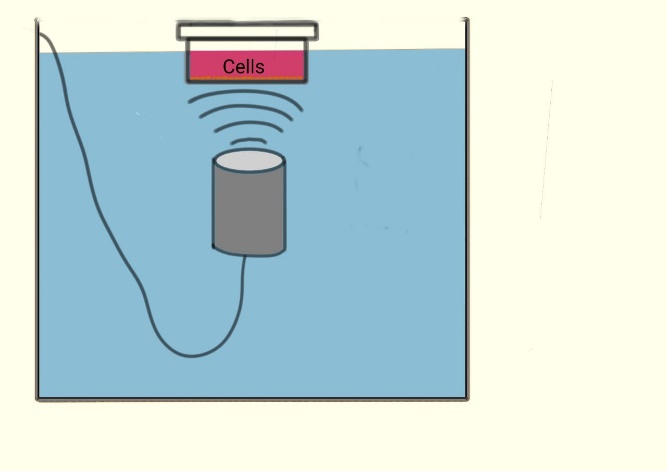
**

**Figure S1. Ultrasound treatment setup for cells within a custom-built water tank.** The cells to be treated were initially seeded on mylar sheets and after 24 hours of incubation in the incubator, the mylar sheets were taken for US treatment.


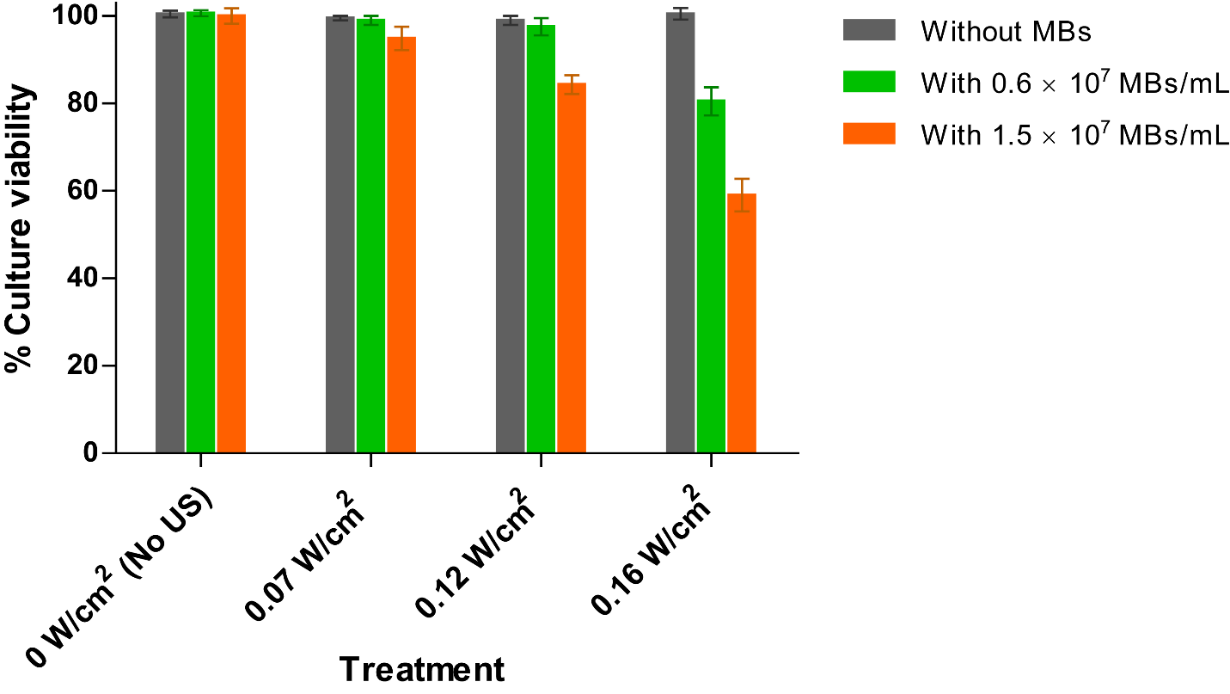


**Figure. S2 Effect of ultrasound/MBs conditions for 100% culture viability.** The MTT assay data with wells treated at different ultrasound intensities of 0.07, 0.12, and 0.16 W/cm^2^ for 15 s in the presence of varying volume ratios of MBs of 1.5×10^7^ (red bar) and 0.6×10^7^ (green bar) MBs/mL of media. The error bars indicate the standard deviation values from triplicate readings for the three different sets of experiments.

**
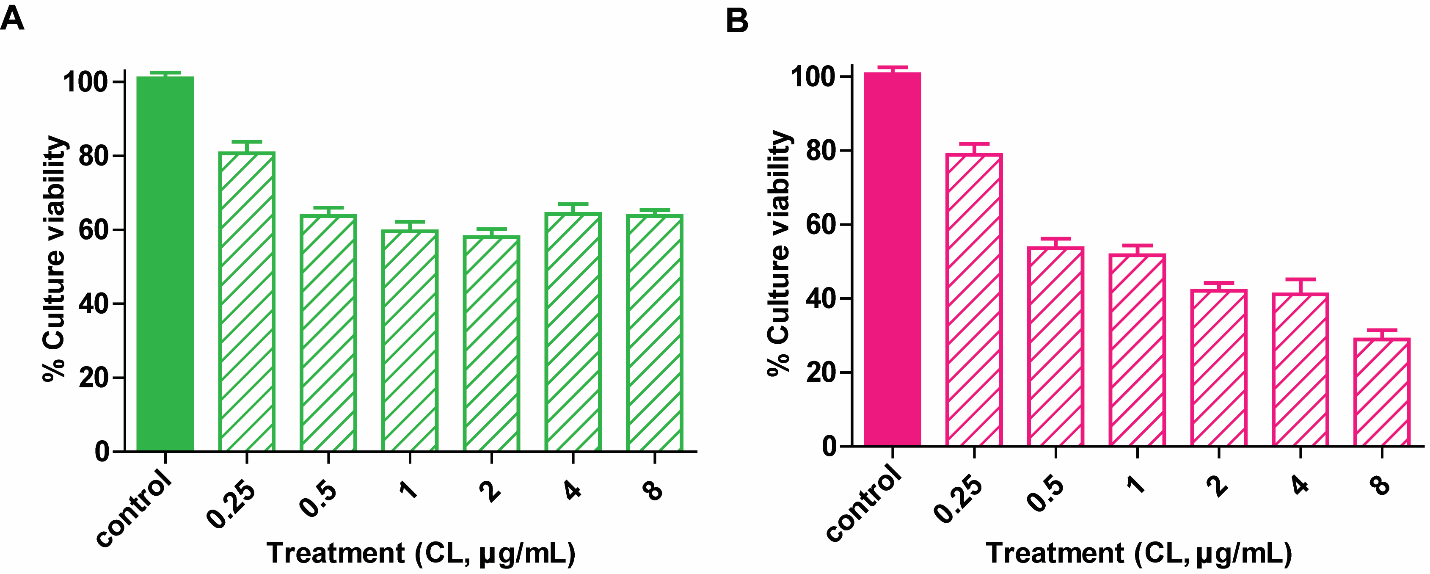
**

**Figure. S3 Effect of treatment time on cytotoxicity assay.** Bar plots indicate the cytotoxicity effect of CLs on HCC 1954 cells when incubated for **A)** 24 hours and **B)** 48 hours during MTT assay**.** The error bars indicate the mean ± standard deviation data of the three different sets of experiments (n=9).
